# Getting the Message Right: Evidence-Based Insights to Improve Organizational Return-to-Work Communication Practices

**DOI:** 10.1007/s10926-021-09961-y

**Published:** 2021-02-02

**Authors:** Arif Jetha, Morgane Le Pouésard, Cameron Mustard, Catherine Backman, Monique A. M. Gignac

**Affiliations:** 1 Institute for Work & Health, 400 University Avenue, Toronto, ON M5G 1S5 Canada; 2 Dalla Lana School of Public Health, University of Toronto, Toronto, ON Canada; 3 University of British Columbia, Vancouver, BC Canada

**Keywords:** Communication practices, Return-to-work, Organizational disability management, Disability managers, Tailoring return-to-work communication, Message framing

## Abstract

*Purpose* There is an absence of evidence-based guidance to support workplace stakeholders in the effective delivery of return-to-work (RTW) messages. Our study examines the specific RTW communication practices and their impact on the management of work disability. *Methods* Within two large and complex healthcare organizations, semi-structured interviews were conducted with workplace stakeholders (e.g., supervisors, union representatives, disability management professionals and workers’ compensation representatives) and workers who had previously experienced sickness absence related to an occupational injury or illness. For workplace stakeholders interview questions asked about their roles and responsibilities in the RTW process, and specific communication strategies and messages that were used at different phases of the RTW process. For worker participants, interview questions explored RTW experiences and the impact of communication on work re-integration. An interpretative descriptive approach was used to inductively examine themes from interviews to create ways of understanding phenomena that yielded applied findings. *Results* Forty participants were interviewed including workplace stakeholders and workers. Participants frequently described effective RTW communication as messages that were delivered by a workplace stakeholder that included the content required by an injured worker to navigate the organizational disability management process and utilized specific strategies to address the perceived attitudes and perceptions held by an injured worker regarding work re-integration. Workplace stakeholders described five specific communication strategies including relaying messages of support, optimizing the timing of communication, careful word choice, framing messages, and tailoring communication to the injured worker. * Conclusion* RTW communication is an active process that requires a strategic approach. Effective communication practices represent an important strategy for workplace stakeholders to address the barriers held by injured workers and foster early and sustained RTW.

## Background

Communication practices are central to organizational disability management. Workplace stakeholders like disability managers, frontline supervisors, and workers’ compensation representatives who effectively convey messages to injured workers can facilitate timely and sustained return-to-work (RTW). Yet, workplace stakeholders often report being ill-equipped to talk to injured workers about disability management and indicate lacking the training necessary to effectively communicate regarding RTW [1]. Also, while early and regular communication with an injured worker is considered best practice in the field of disability management, there remains an absence of evidence-based resources that guide workplace stakeholders on the specific ways in which RTW messages ought to be delivered [2–4]. The importance of effective and consistent messaging to injured workers is especially important within large and complex organizations where variable RTW outcomes may be more common [5]. Within two large healthcare organizations, our qualitative study examines specific RTW communication practices and their impact on the management of work disability. Results offer strategies that can be utilized by workplace stakeholders to effectively communicate with injured workers during RTW.

Workplaces are social environments where effective communication can help to establish trust between workers and management and effectively deliver human resource practices [4, 6–9]. In the field of disability management, communication has frequently been discussed as playing an important role in facilitating RTW of workers who experience sickness absence attributed to an occupational injury or illness [1, 10, 11]. A well-established practice has been to encourage workplace stakeholders to initiate early contact with an injured worker and maintain regular communication to improve RTW outcomes [4, 8]. Also, the development and implementation of disability management plans are more effective when workplace stakeholders react favorably to a worker reporting an injury or illness, continues to deliver messages of reassurance over the RTW process, and conveys practical details regarding organizational policies and processes to an injured worker [3, 11–13]. A recent study of workers’ compensation claimants (n = 869) in Victoria, Australia showed that participants who received supportive messages from their supervisors at the time of an injury had a 2.3 and 1.6 times greater likelihood of reporting sustained RTW at baseline and after 6 months, respectively [14]. Other research shows that communicating messages of support early and over the course of the RTW process has the potential to encourage the reintegration of injured workers [8]. To date, limited information exists for workplace stakeholders on best practices for communicating with injured workers and delivering messages of support during RTW. Additional evidence-based guidance on communication can enhance organizational disability management practices and outcomes.

The effective communication of workplace stakeholders is especially important within large organizations where employees may be situated across multiple departments and where job demands and responsibilities can vary [1]. Research conducted within large organizations shows that disability management is more likely to be coordinated by multiple stakeholders inside and outside of an organization [1]. Additionally, within large organizations, inconsistent training on work disability management practice, inharmonious relationships between workers and supervisors, and different levels of experience with RTW can contribute to communication challenges [1, 10, 15, 16]. Importantly, gaps in communication and delays of information exchange can have a ripple effect across large organizations and contribute to delays in RTW [16]. Research on communication practices at the organizational level has the potential to provide workplace stakeholders with strategies to improve coordination and delivery of information to injured workers.

Behavioral science and health communication theory can be utilized to enhance research and practice by informing our understanding of the strategies that workplace stakeholders could use to customize messaging and facilitate RTW behaviors [17–19]. Through the lens of these theoretical perspectives, delivering messages that address the specific attitudes an injured worker holds towards re-entering work (e.g., preparedness, normative beliefs, evaluation of RTW outcomes) and their perceptions regarding RTW obstacles in the work environment (e.g., feelings of controls) can encourage preparedness and the behaviors necessary to re-enter work (e.g., health-seeking, rehabilitation, RTW planning, re-entry and stay-at-work) [20, 21]. Drawing from health communication theory, adjusting the content and timing of RTW messages to an injured worker’s characteristics and stage of recovery can ensure that messages are most relevant to a worker and ensure that they are more likely to contribute to the behavioral changes necessary for work reintegration [22, 23]. To date, the application of behavioral science and health communication theory to better understand how RTW communication practices can impact organizational disability management remains limited.

The aim of our research was to examine the role of communication practices in the work disability management process within two large and complex healthcare organizations and fill gaps identified by theory and research in behavioral sciences and health communication. In particular, we conducted a qualitative study to address two specific research objectives: (1) To elaborate on the content of communication between a workplace stakeholder and its impact on the attitudes, perceived barriers and RTW behaviors of injured workers; (2) To describe specific communication strategies that were used by participants to facilitate RTW. Insights developed from this study have the potential to be used by workplace stakeholders to improve RTW communication practices.

## Methods

### Disability management context

Our study was set within British Columbia’s healthcare system, which is supported by over 115,000 employees that are based in seven health authorities and operate in acute (e.g., hospitals), long-term care (e.g., nursing homes) and community healthcare settings (e.g., home care) [24]. Data collection was conducted within two specific health authorities. Both healthcare organizations provided care to over 1,050,000 people across large geographic regions [25]. Within both healthcare organizations, RTW of occupational injury or illness is administered according to a collectively bargained disability management program that is implemented in collaboration with the injured worker and a team of workplace stakeholders that include the immediate supervisor and disability management professional (employed by the healthcare organization), an external union representative and a representative from the provincially legislated worker’s compensation system. Holistic disability management plans are developed collaboratively to set milestones and expected outcomes and determine specific work re-integration activities including access to medical intervention and rehabilitation, development of transitional work opportunities, graduated RTW, workplace modification and vocational rehabilitation and/or retraining. RTW plans are based on an assessment of factors such as prognosis, capabilities, limitations, and evaluation of workplace factors (e.g., extent to which job tasks are modifiable) [26]. The disability management program used by each healthcare organization was generated through specific recommendations from a comprehensive evaluation of RTW practices within British Columbia’s healthcare system conducted in 2008 [27]. Of note, within participating healthcare organizations, disability management of workers with non-occupational injury or illness were supported by a different set of policies and procedures. For this study, we choose to focus specifically on the experiences of workers with occupational injury or illness to capture the impact of a collectively bargained disability management program on communication practices and explore ways in which messages were delivered by a diverse set of RTW stakeholders.

### Participant Recruitment

Within the two healthcare organizations we interviewed workplace stakeholders (e.g., frontline supervisors, union representatives and disability management professionals and workers’ compensation representatives) and workers who had previously experienced sickness absence related to an occupational injury or illness. Recruitment was facilitated by a workplace representative who circulated study invitations to potential participants through a general email. Interested participants were asked to contact a member of the research team directly for study and consent information and to schedule an interview. Snowball sampling was used to identify additional participants within an organization and to address emerging themes. To capture depth and breadth in our understanding of RTW communication, participant recruitment occurred until no new themes emerged from the interviews [28]. All procedures were approved by the research ethics boards of the University of British Columbia (REB# H19-00324) and University of Toronto (REB# 37205).

### Interview Approach

Within each healthcare organization, interviews were semi-structured, primarily face-to-face and lasted one hour. For workplace stakeholders interview questions asked about roles and responsibilities in the RTW process, and specific communication strategies and messages that were used at different phases of the RTW process. Follow-up questions probed for perspectives on the impact of communication practices on RTW, and how messages were tailored to different worker characteristics. For worker participants, interview questions explored RTW experiences and the impact of communication on work re-integration attitudes and behaviors and outcomes. All participants provided informed consent and interviews were held in private meeting rooms. Participants were provided the option to conduct interviews over the phone for additional confidentiality.

### Data Analysis

All interviews were audio-recorded and transcribed verbatim. To build on an understanding of RTW communication, we took a constructivist epistemological perspective to consider knowledge and meaning that is produced from a research participant’s personal experiences [29, 30]. Several steps were taken to apply our analytical approach. Members of the investigative team independently reviewed transcripts to develop an initial codebook. Utilizing the codebook, two members of the research team separately coded a workplace stakeholder and worker transcript to ensure codes were applied consistently to the data. The codebook was refined through discussions and applied to the analysis of the remaining transcripts. To ensure dependability of the findings, two independent coders conducted line-by-line coding of each of the remaining transcripts. Coding was compared and discrepancies were reconciled in face-to-face meetings. Members of the research team had experience in conducting qualitative research and/or were subject matter experts in the field of disability management. Once coding of transcripts was complete, prominent themes were extracted using an interpretative descriptive approach where themes were inductively analyzed to create ways of understanding phenomena that yielded applications within the organizational disability management program [31, 32]. Themes from workplace stakeholders and workers were contrasted to understand the communication strategies that could facilitate or constrain RTW. Throughout the analytical process, codes and themes selected from the data were discussed in analysis meetings with the investigative team. Any inconsistencies were resolved by consensus. Salient themes that were identified by the analytical process were member-checked through consultations with workplace partners from each organization to ensure confirmability. Analysis was facilitated by using the software NVivo [33].

## Results

Forty participants were interviewed consisting of disability management professionals (25%), supervisors (35%), union representatives (12%), workers’ compensation representatives (8%) as well as workers (20%) who had previously experienced an occupational injury or illness and participated in the RTW process. Consistent with the gender make-up of British Columbia’s healthcare sector, most participants in our sample were women (85%). Participants frequently described effective RTW communication as messages that were delivered by a workplace stakeholder that possessed the content required by an injured worker to navigate the organizational disability management process and utilized specific strategies to address perceived attitudes and perceptions held by an injured worker regarding work re-integration. In the following sections, we provide greater insight into effective communication practices.

### RTW Communication Content and Information Exchanges

A combination of verbal (e.g., telephone) and written (e.g., email, forms) communication was central to disability management practices within both healthcare organizations. At its core, RTW communication was seen as a two-way transactional process between workplace stakeholders and workers. The information initially exchanged from workers to workplace stakeholders included medical (e.g., injury details, functional limitations) and administrative information (e.g., recovery duration, accommodation information) that was needed to initiate and implement a disability management plan. The information exchanged from workplace stakeholders to workers included policies and procedures (e.g., details on the disability management program, workers’ compensation information) and roles and responsibilities (e.g., role of different workplace stakeholders in coordinating RTW). Consistent information exchange was central to the implementation of organizational-wide disability management. For example, a disability management professional described their organization’s approach to exchanging information to an injured worker during the RTW process:

“…Initially, giving them [injured worker] a call, providing an introduction in terms of who you are… ‘The purpose of the program is to ensure that you have support and services and to assist with your timely recovery and return’… ‘Would you mind telling me a little bit about your recent injury in the workplace? Some information on what your current abilities are or the recommendations by your doctor’. Then asking questions and providing them with the information to reaffirm for them that it’s a safe process.” – *Disability management professional (DM1)*

Similarly, a workers’ compensation representative noted that many injured workers who engaged in RTW are confused about the roles and responsibilities of different workplace stakeholders. The workers’ compensation representative described using information sheets as a specific strategy to distil key messages to injured workers to foster information exchange:

“…I developed cheat sheets, I guess you’d call them, which were just basic. ‘This is your disability case manager, and this is what your disability case manager helps with. I am your vocational rehabilitation consultant. This is what I help with. These are the decisions that happen on your claim’” **–** *Workers’ compensation representative (WC1)*

At the same time, the content of communication and exchange of information was sometimes seen as being affected by several factors including privacy policies and trustworthiness of the source. Human rights legislation, workplace policies and collectively bargained disability management policies included privacy protection for injured workers. These policies coupled with a worker’s potential personal preferences to not communicate all of the details of their injury or illness represented a frequently described barrier to exchanging information. One disability management professional, talked about how challenges of collecting information could affect their ability to respond to a worker’s needs when developing and implementing RTW plans:

“… If people are keeping the reasons why they’re staying away from work close to their heart and they don’t want to tell you or they don’t want to share with you, you can’t give them the information that they might need to resolve those issues.” – *Disability management professional (DM2)*

Also, to facilitate information exchange, workplace stakeholders frequently described the importance of building trust. They also talked about workers needing to feel comfortable with being asked about their health and sharing their perceptions regarding work re-entry. One participant, who was a disability management professional, talked about the importance of utilizing the first conversation with a worker following an injury to ask key questions needed to develop the RTW plan and build trust:

“…it’s also important to get the employee’s perspective because some of the questions that they [disability management professional] are asking are things like, ‘what happened?’ But also, in their [injured worker] work duties what do they think will be challenging when they return to work, what concerns them about returning to work? What concerns them about not returning to work? So, getting the employee’s perspective right off the bat.” – *Disability management professional (DM3)*

However, not all workplace stakeholders were seen by injured workers as trustworthy and information was not shared equally across all individuals responsible for disability management. Worker participants in our study were more likely to report that their union representatives were seen as the most trustworthy stakeholder with whom they could provide a greater number of details on RTW progress and discuss specific challenges to work re-entry. For example, a union representative participant noted that being perceived by injured workers as a trusted source meant that they could more effectively deliver information to injured workers to address the specific challenges they might face as they progressed through RTW:

“All of the stakeholders walk in with their own agenda because they need to get from point A to point B to point C and sometimes, I feel that the employee is kind of left in the shuffle and even when some of the things that they want are unreasonable, I think they should still be thought out so that the employee realizes, okay, they are listening to me” – *Union representative, UR1*

On the other hand, certain workplace stakeholders (e.g., supervisor, disability management professional) were less likely to be perceived as trustworthy by workers. Accordingly, some supervisors and disability managers who participated in our study reported experiencing challenges exchanging information and as a result faced limitations to collecting the details needed to plan and implement RTW. One disability manager described their response to a worker who expressed a lack of trust and hesitancy in sharing RTW details and described the importance of continued engagement with union representatives.

“I can say, ‘I understand you’re feeling a bit guarded about the information, I want to reassure you that I am to going have the union disability management rep follow-up with you just to reassure you of whatever you need reassurance on’, and then I’ll follow-up next week…I try to find what feels comfortable for them, and it [hesitancy to talk about an injury] happens all the time in this role” – *Disability management professional (DM4)*

### RTW Communication Strategies

Participants talked about five categories of strategies that were utilized to effectively communicate messages regarding RTW and that were aimed at helping workers with RTW. Strategies included communicating messages of support, optimizing the timing of communication, carefully wording messages, framing messages and tailoring communications (Table 1).

**Table 1:** Summary of communication strategies highlighted by workplace stakeholders responsible for disability management and workers who had previously experienced an occupational injury or illness in two large healthcare organizations

Strategy	Description	Examples described by participants
Communicating messages of support	Accompanying information with messages of instrumental and emotional support	Offering consistent messages of encouragement to a worker across phases of return-to-work (RTW)
RTW communication timing	Initiating early conversations with an injured worker and sustaining regular communication across all stages of RTW	Building rapport with a worker following an injury Scheduling regular status updates, offering feedback and asking probing questions to assess worker’s recovery and readiness
Carefully wording messages	Adapting the delivery of messages to facilitate comprehension and explain technical or legal concepts in a disability management plan	Simplifying the language used in conversations Breaking down and describing each step in the RTW process for a worker Using written communication to reinforce verbal correspondences
Framing messages	Constructing messages that may emphasize the benefits of returning to work or the downsides of a prolonged absence	Highlighting the psychological and physical benefits of RTW Describing financial implications or mental health consequences that can be associated with not returning to work
Tailoring messages	Selecting communication strategies that are tailored to the personal characteristics of the injured worker and their perceptions regarding RTW	Adjusting RTW communication strategies used by a workplace stakeholder to a worker’s injury characteristics, perceived RTW readiness or their comprehension of the disability management plan

#### Communicating Messages of Support

Working at a large organization, workplace stakeholders often noted that workers held varying attitudes and perceptions regarding RTW. Some workers were highly motivated to re-enter work after an injury. Others were more fearful or perceived a greater number of barriers to RTW. To help a worker navigate through each step of the RTW process, workplace stakeholders talked about the need to accompany information with messages of support. One participant who was a supervisor reflected on the experience of providing support while also offering tangible assistance in RTW. They described messages of support as:

“Genuinely asking ‘how are you doing?’ ‘Is there anything I can help you with?’ ‘Is there anything you need?’ ‘Is the plan working for you?’ ‘Do we need to do anything different?’ Making sure that they are with somebody that can keep an eye on them.” – *Supervisor (S1)*

On the other hand, a worker participant, described how the absence of support and a clearly described RTW plan left them feeling alienated  from their work environment during sickness absence.

“In this type of case, it would be having a plan for me, welcoming you back. Even if you fake it. It was clear at the end when I was there, I would say, I don’t think she (supervisor) wants me back…As far as disability management, I didn’t feel much support there at all.” – *Worker (W1)*

Communicating support meant that participants were also providing messages of encouragement to a worker through the RTW process. Workplace stakeholders provided different examples of messages of encouragement they relayed to injured workers, including emphasizing the importance of the injured worker to the immediate team and to meeting organizational goals and expressing enthusiasm regarding work re-entry. A union representative noted that offering consistent messages of encouragement was especially important to communicating support in prolonged or complex disability management cases. They described an example of providing encouragement in their conversation with an injured worker:

“… ‘This is going to be okay’, and just a lot of, ‘you’re going to do great’, and encouragement of, ‘I know you can do this, I know you can do this. If it doesn’t work, it’s all right, we’ll figure out plan B next. But give it a try, I know you can do this.’”**-** *Union representative (UR2)*

Of importance, workers and workplace stakeholders noted that the absence of supportive messages contributed to RTW delays. From their experience, a participant who was a workers’ compensation representative, discussed how a lack of support and infrequent communication could lead to feelings of isolation from an injured worker:

“I’ve had workers that felt completely forgotten and sort of hurt by employers that, probably through no fault of their own, they just didn’t feel it was appropriate to contact the person while they were off injured or whatever. But the perception from the injured worker is that they’ve kind of lost that place. Somebody may have worked there for 30 years and the day that they left when they got hurt is the last they heard from the employer…” – *Workers’ compensation representative (WC2)*

#### RTW Communication Timing

The element of time frequently emerged in interviews with workplace stakeholders as being a critical RTW communication strategy. Aligning with an organizational policy of early contact, participants described the importance of initiating conversations with an injured worker so that they could offer details about the RTW process and provide support to the injured worker. Initial conversations were central to setting the tone for RTW and subsequent conversations. For instance, a disability management professional described the initial conversation as an important opportunity to build relationships and begin information exchange.

“That building rapport is key. Right from the very beginning. The very first conversation. Start it off the way you want it to carry forward. And in that building of rapport, that you’re letting them know what your role is and isn’t, so that they are aware what I can help them with and not be disappointed if I can’t help with something.” – *Disability management professional (DM5)*

Similarly, a worker participant, described the initial conversation as an important opportunity to build relationships with a workplace stakeholder regarding RTW and begin exchanging information.

“It comes to communication, opening a dialogue, having a conversation and offering support is huge, just being able to know that they [workplace stakeholder] understand and that they are willing to help in any way that they can.” – *Worker (W2)*

In addition to early contact following an injury, regular follow-up communication with injured workers were described by workplace stakeholders as critical to implementing RTW plans, monitoring RTW progress, and reinforcing support. Given the diversity of recovery experiences, workplace stakeholders acknowledged that follow-up conversations were not always linear, and that communication could often be a back-and-forth process. Accordingly, workplace stakeholders frequently talked about the importance of identifying creative ways to repeat and reiterate the details of a disability management plan. A participant who was a workers’ compensation representative described the importance of asking a range of different questions as a strategy to better understand and probe worker’s status in the recovery progress and their perceptions of readiness to re-enter work. They noted that:

“What we train is that the conversations are never linear, and certainly the first conversation isn't. It might go in all kinds of different directions and that will get the answers that you need but it isn't a question and answer, it's a discussion. And we do ask about, how are things at home, how are you managing, are things okay, what's difficult for you, are there hobbies that you can't participate in right now because of your injury and how does that feel? So, we have discussions rather than an interview.” – *Workers’ compensation representative (WC3)*

Over the course of a prolonged disability case, workplace stakeholders also talked about other strategies used to sustain communication including scheduling regular check-ins, offering feedback and providing RTW reminders, and delivering messages that reiterated organizational policies.

#### Carefully Wording Messages

Another RTW communication strategy frequently described by participants was the importance of carefully choosing the rights words. Both workers and workplace stakeholders often described much of the RTW documentation and information delivered to workers following an injury as being technical and legal. Word choice was a critical element related to a worker’s comprehension of messaging. Workplace stakeholders described the importance of simplifying language, providing time for participants to absorb information during a conversation, breaking down components of the RTW process for a worker, and using illustrative stories to highlight the importance of work reintegration. A disability management professional participant, noted that:

“I tend to not use a lot of jargon or usual terms. I tend to use layman’s terms, as if I’m having a conversation with someone that I know pretty well. It kind of eases the way into it” – *Disability management professional, DM6*

Similarly, workers talked about the importance of receiving carefully worded written communication to reinforce messaging delivered during phone conversations with a workplace stakeholder. This was especially important for workers whose injury may have affected cognition or attention. For instance, a worker participant who had suffered a brain injury, talked about the benefits of receiving a simple breakdown of steps in the RTW process to supplement verbal correspondences. They noted that that:

“They [workplace stakeholder] always sent me written summaries afterwards. Everybody would do that. [Disability management professional] would send me this is [referring to written communication]… like, so we are going to do your return-to-work and then they sent me the schedule. She told me what was expected of me. The concussion specialist literally sent me a huge list of things that I was expected to do and expected not to do… So that was really helpful. She was great at communicating that plan.” – *Worker (W3)*

#### Framing Messages

Communication style was also marked by the framing of RTW messages. In their conversations with injured workers, workplace stakeholders often reported delivering messages that either emphasized the benefits of RTW or highlighted the downsides to not returning to work. The framing of messages often depended on the duration of absence. At the early phase of a work disability case, workplace stakeholders commonly described that messages they provided to an injury worker highlighted the benefits of work re-entry (e.g., potential health, psychosocial or financial benefits of RTW) in order offer encouragement. A disability management professional noted that framing the benefits of RTW tended to resonate with most workers within the healthcare organization in which they worked:

“My personal belief is that the most motivating is when you highlight what the benefits are to that individual. And, I mean, that’s to the workers as well as the manager. ‘Why should you be invested in this process?’ Well, for a worker, you need to ensure your source of income, your job is part of your identity, it feels good to be at work, it provides you with purpose, it will prevent further deconditioning and additional medical complications, you’ll maintain your benefits. We make a conscious effort to intertwine the organization’s values. We’ve got empathy, respect, collaboration, and innovation. So, I like to highlight those in relation to our work as well, because I truly believe that most of our people are invested in being people that live our values.” – *Disability management professional (DM1)*

Workplace stakeholders who participated in our study described that they were less likely to highlight the negative implications of sickness absence to an injured worker. However, in more prolonged absences, workplace stakeholders described discussing the downsides to not re-entering work as a communication strategy of last resort. A study participant who was a supervisor reflected on conversations with an injured worker who was resistant to engaging in RTW.

“…Just having those conversations and putting it more into a financial aspect and how that would impact her [injured worker], it made more sense to her. So, again, she was resistant, but she did come back in…I always go the financial route, because some people can, and some people can’t and know your employees… Sometimes that’s a little bit more of a motivator.” – *Supervisor (S2)*

Other examples of messages delivered by workplaces that described the implications of prolonged sickness included those that emphasized negative mental health implications or the increasing RTW difficulties that can be attributed to a prolonged absence.

#### Tailoring Communication

To successfully deliver messages, workplace stakeholders consistently talked about the importance of tailoring their communication strategies (i.e., messages of support, communication timing, choosing the right words, message framing) to the specific injured worker to which they were speaking. In particular, participants talked about tailoring communication strategies according to the characteristics of an injured worker (e.g., age, educational level, job role) and their readiness to RTW, communication style and level of comprehension of the disability management plan. An active and iterative process was utilized by workplace stakeholders to develop tailored messages. For instance, a disability management professional participant highlighted the importance of disability managers being able to read the cues of the worker and tailor their communication style, accordingly. The participant noted that:

“ They [RTW messages] are tailored to the individual. They’re [disability managers] fairly good at reading cues via phone, in terms of where they might need to give the person a bit more time before they dive into details, versus those that are open communicators and don’t have any additional barriers or assumptions in regard to the program”. – *Disability management professional (DM1)*

As an example, another disability management professional participant described tailoring the amount of information and tone of the conversation based on the characteristics of the worker. They noted that:

“A lot of times you have to take cues from the person that you’re talking to in terms of how much of the tone is supportive versus how much of it is sharing information. How much information you are going to share in that initial contact, sometimes you are contacting employees who are quite distressed and maybe not able to hear all of the information, it’s too much for them. You have to gauge depending on who you are talking to, but the tone needs to be supportive, but they also need to know that the goal is for early safe return-to-work.” – *Disability manager professional (DM3)*

Tailoring messages was frequently described by workplace stakeholders as an important approach to increase the uptake of information to facilitate RTW. Worker participants often noted that when communication was unique to their circumstances, they felt that they were being approached from a place of concern. However, when a message was more generic and appeared scripted, they perceived the workplace stakeholder as less concerned about their recovery and reported less motivation to RTW. When reflecting on their RTW experience, a worker participant noted that:

“…I think it’s like they’re reading a script. That’s how it came across or how it was presented. It’s like ‘mmm’ but there’s no ‘oh hi’ almost monotones… It’s like they’re just trying to do their job, get the information and get out…I think you’re better off to call a spade a spade and deal with that person individually and move in. Instead of painting everybody with the same brush.” – *Worker (W3)*

Similarly, other worker participants consistently described that when communication was tailored to their health and personal situation it was seen as more effective in fostering RTW.

## Discussion

Communication is central to the successful development and implementation of organizational disability management policies and procedures. While workplace stakeholders are frequently asked to effectively communicate with injured workers, there exists few evidence-based recommendations on the specific strategies to deliver information about RTW. Our research, conducted within two large healthcare organizations, fills critical applied knowledge gaps. We provide an in-depth view of the characteristics of effective RTW communication that address the attitudes and perceptions held by injured workers as they engage in the organizational disability management process. Findings highlight approaches for effective RTW communication including focusing on the timing of messages, choosing the right words, and framing messages. Of importance, tailoring messages to the characteristics of participants was seen as central to RTW communication and could directly address RTW perceptions held by an injured worker and promote the behaviors necessary for work reintegration. Our findings can be used by organizations to strengthen RTW communication practices.

Within the two healthcare organizations in which data was collected, communication was consistently seen as a two-way exchange of information. Our findings add to the research which shows the importance of communicating the practical details to navigate workers through a disability management plan [11, 34]. Providing practical details about organizational policies and procedures to injured workers can be especially important within large organizations where RTW can be more complex [1, 16]. Of significance, findings from our study indicate that the exchange of information in conjunction with messages of support can be a particularly effective communication strategy that can foster RTW. Results align with growing evidence on the importance of the social work environment and delivering social support to successful RTW [1, 3, 7, 8, 35]. Drawing from theoretical literature in the behavioral sciences, messages of support offered by workplace stakeholders can address the perceived barriers that an injured worker may hold towards RTW and provide encouragement that is needed to foster RTW readiness and work re-integration behaviors [20, 36]. It is important to acknowledge that within the context of a collectively bargained work disability management program, not all workplace stakeholders may be seen as trustworthy. Future research is needed to elaborate on how messages delivered by different workplace stakeholders are perceived by injured workers and their impact on RTW behaviors. Nonetheless, findings point to the importance of different workplace stakeholders responsible for work disability to establish trust and credibility with an injured worker in order to maintain communication during RTW [7].

The timeliness of messages was also found to be an important communication strategy. Study participants consistently described RTW as a non-linear process where communication between a workplace stakeholder and injured worker occurred over multiple time points. Workplace stakeholders in our study emphasized that early contact with injured workers was necessary to begin exchanging the information needed to establish an RTW plan, build rapport and set the tone for subsequent conversations. Our findings can be contextualized within established practice in the field of work disability management that highlight the importance of early communication following occupational injury to facilitate RTW, increase worker engagement in the disability management and ensure the ultimate success of work reintegration [3, 37–39]. Our study is novel in that it highlights the importance of sustained communication over the duration of a disability episode. Workplace stakeholders in our study elaborated on the non-linearity and unpredictability in more prolonged RTW cases. In response, many participants acknowledged that a dynamic and creative approach to sustaining RTW communication is necessary. Maintaining effective communication practices represents an important skill for workplace stakeholders responsible for disability management. Organizations should provide training to workplace stakeholders on how to reinforce communication over the course of a disability episode. Additionally, to build on our study findings, more attention should be directed towards understanding the specific communication strategies that can be used by workplace stakeholders to reinforce sustained work integration once a worker has successfully returned to work.

Along with the timeliness of messages, participants also acknowledged the importance of carefully selecting words they used in their conversations with injured workers. As described in previous studies, the planning and implementation of RTW has a number of legal and technical components that can be a source of confusion for injured workers and contribute to delays in work reintegration [1, 11]. To improve comprehension, workplace stakeholder participations discussed the importance of simplifying language in correspondences, providing time for an injured worker to absorb information, breaking down key steps in the RTW process, providing follow-up communication in writing, and using illustrative stories to highlight the implications of RTW. In our qualitative study, there was not one specific strategy that was ranked by participants as being most effective in improving comprehension. Accordingly, our results show that a one-size-fits-all approach to delivering RTW messages may not be effective. Workplace stakeholders should continue to view RTW communication as an active process that requires a strategic approach. Research conducted in a larger sample spanning different industries could help to develop a better understand of the worker cues or characteristics that precede a workplace stakeholder making adjustments to their word choice.

Messages were often framed by workplace stakeholders as a strategy to encourage worker participation in RTW. In most cases, workplace stakeholder described that framing the various benefits of RTW to an injured worker was preferable and tended to be an effective approach within the healthcare organizations in which they worked. However, in more prolonged disability episodes, participants noted framing RTW messages that emphasized the negative implications of a delayed RTW. Findings can be conceptualized within the framework of prospect theory, which has been applied to studies to inform the delivery of health messages. Prospect theory posits that health messages can be expressed with respect to their associated costs (loss-framed) or benefits (gain-framed) [22, 23, 40]. According to the theory, individuals may be more willing to accept risks of a target behavior when options are framed in terms of related costs. In contrast, people may avoid risks when the same options are framed in terms of related benefits [22]. Applied to different health behaviors (e.g., smoking, physical activity, cancer screening), studies utilizing prospect theory have compared whether individuals respond more favorably to different message frames as a way to inform the design of communication-based interventions [23, 40–43]. Within the healthcare organizations in which data was collected, our study shows that framing the benefits of RTW may be most effective to promote behavioral response in most disability management cases. Loss-framed messages may be a message-frame of last resort. Additional research with larger samples across more diverse industries is required to examine the impact of different message frames on diverse outcomes including RTW readiness, absence duration, work re-entry and sustained RTW.

A body of previous research shows that RTW is a highly variable process that can differ according to a system of personal, health, and contextual factors [11, 16]. Our study results indicate the importance of tailoring communication strategies to an injured worker and their injury experiences. Research conducted in the field of health communication show that delivering messages that are unique to the recipient are significantly related to changing attitudes and promoting behavioral activation [44, 45]. Growing from health communication research, our findings provide preliminary support for the tailored messages to address perceived barriers to RTW held by an injured worker and to encourage readiness to RTW. To advance organizational disability management practices, our study points to the need for training and coaching to workplace stakeholders on how to adjust communication practices to individual workers. Subsequent quantitative research are required to further examine how tailored messages delivered to injured workers can impact the length of a disability episode and organizational RTW outcomes.

Our study is one of the first to delve into specific communication strategies that can be utilized by workplace stakeholders to facilitate RTW within large and complex organizational settings. Also, our inclusion of diverse workplace stakeholders responsible for disability management and workers who had previously experienced RTW enabled us to capture a range of communication practices. Our inclusion of diverse study participants also enabled us to determine similarities and differences in the RTW messages delivered by workplace stakeholders and their impact on the attitudes and behaviors held by injured workers. Importantly, the measures taken in our study to address trustworthiness (e.g., member-checking results) suggests that results could be transferable to other large and complex healthcare organizations. It is important acknowledge that missing from our study was participants who were unable to successfully RTW. To build on this limitation, research is required to understand how communication practices could have contributed to unsuccessful RTW attempts for injured workers.

## Conclusion

Communication between a workplace stakeholder and injured worker plays an important role in determining organizational disability mangement outcomes. Our study fills an important knowledge and practice gap by identifying the strategies taken by workplace stakeholders to effectively communicate regarding RTW. Specific strategies highlighted in our research include coupling information exchange with support, appropriately timing RTW messages, choosing appropriate wording, and balancing gain- and loss-framed RTW messages. Above all, tailoring communication strategies to an individual worker and their work context could be especially important in facilitating RTW. Results from our study have practical implications for workplace stakeholders responsible for RTW and communicating with injured workers.

## References

[1] Jetha A, Yanar B, Lay AM, Mustard C (2019). Work disability management communication bottlenecks within large and complex public service organizations: a sociotechnical systems study. J Occup Rehabil.

[2] Franche R-L, Severin CN, Hogg-Johnson S, Côté P, Vidmar M, Lee H (2007). The impact of early workplace-based return-to-work strategies on work absence duration: a 6-month longitudinal study following an occupational musculoskeletal injury. J Occup Environ Med.

[3] Linton SJ, Boersma K, Traczyk M, Shaw W, Nicholas M (2016). Early workplace communication and problem solving to prevent back disability: results of a randomized controlled trial among high-risk workers and their supervisors. J Occup Rehabil.

[4] Franche R-L, Cullen K, Clarke J, Irvin E, Sinclair S, Frank J (2005). Workplace-based return-to-work interventions: a systematic review of the quantitative literature. J Occup Rehabil.

[5] Jetha A, Pransky G, Hettinger LJ (2016). Capturing complexity in work disability research: application of system dynamics modeling methodology. Disabil Rehabil.

[6] Argenti PA (1998). Strategic employee communications. Hum Resour Manag.

[7] Friesen MN, Yassi A, Cooper J (2001). Return-to-work: the importance of human interactions and organizational structures. Work.

[8] White C, Green RA, Ferguson S, Anderson SL, Howe C, Sun J (2019). The influence of social support and social integration factors on return to work outcomes for individuals with work-related injuries: a systematic review. J Occup Rehabil.

[9] Gignac MA, Jetha A, Martin Ginis K, Ibrahim S (2021). Does it matter what your reasons are when deciding to disclose (or not disclose) a disability at work? The association of workers’ approach and avoidance goals with perceived positive and negative workplace outcomes. J Occup Reabil..

[10] Gignac MA, Bowring J, Jetha A, Beaton DE, Breslin FC, Franche RL (2020). Disclosure, privacy and workplace accommodation of episodic disabilities: organizational perspectives on disability communication-support processes to sustain employment. Generations.

[11] MacEachen E, Kosny A, Ferrier S, Chambers L (2010). The, “toxic dose” of system problems: why some injured workers don’t return to work as expected. J Occup Rehabil.

[12] Post M, Krol B, Groothoff J (2005). Work-related determinants of return to work of employees on long-term sickness absence. Disabil Rehabil.

[13] Gray SE, Sheehan LR, Lane TJ, Jetha A, Collie A (2019). Concerns about claiming, postclaim support, and return to work planning: the workplace's impact on return to work. J Occup Environ Med.

[14] Jetha A, LaMontagne AD, Lilley R, Hogg-Johnson S, Sim M, Smith P (2018). Workplace social system and sustained return-to-work: a study of supervisor and co-worker supportiveness and injury reaction. J Occup Rehabil.

[15] Collie A, Newnam S, Keleher H, Petersen A, Kosny A, Vogel AP (2019). Recovery within injury compensation schemes: a system mapping study. J Occup Rehabil.

[16] Jetha A, Pransky G, Fish J, Hettinger LJ (2015). Return-to-work within a complex and dynamic organizational work disability system. J Occup Rehabil.

[17] Brouwer S, Krol B, Reneman MF, Bültmann U, Franche R-L, van der Klink JJ (2009). Behavioral determinants as predictors of return to work after long-term sickness absence: an application of the theory of planned behavior. J Occup Rehabil.

[18] Dunstan DA, Covic T, Tyson GA (2013). What leads to the expectation to return to work? Insights from a theory of planned behavior (TPB) model of future work outcomes. Work.

[19] Bültmann U, Brouwer S (2013). Individual-level psychosocial factors and work disability prevention. Handbook of work disability.

[20] Ajzen I, Kuhl J, Jürgen B (1985). From intentions to actions: A Theory of planned behaviour. Action control from cognition to behaviour.

[21] Gallagher KM, Updegraff JA (2012). Health message framing effects on attitudes, intentions, and behavior: a meta-analytic review. Ann Behav Med.

[22] Rothman AJ, Salovey P (1997). Shaping perceptions to motivate healthy behavior: the role of message framing. Psychol Bull.

[23] Corcoran N. Chapter 1: Theories and models in communicating health messages. In: Communicating health: strategies for health promotion. London: Sage Publishing; 2007. p. 5–31.

[24] Health Employers Association of BC. Health care workers in BC Vancouver2014. Available from: http://www.heabc.bc.ca/Page4337.aspx#.Wk5ZfhM-fOT.

[25] Interior Health Authority. About us Kelowna, BC2017. Available from: https://www.interiorhealth.ca/AboutUs/.

[26] Health Employers Association of BC. Enhance Disability Management Program (EDMP) policies and procedures 2014. Available from: http://www.heabc.bc.ca/public/edmp/PoliciesandProceduresCBA.pdf.

[27] National Institute of Disability Management and Research. Disability management and organizational change: the disability management action research project. Port Alberni BC; 2011.

[28] Varpio L, Ajjawi R, Monrouxe LV, O'Brien BC, Rees CE (2017). Shedding the cobra effect: problematising thematic emergence, triangulation, saturation and member checking. Med Educ.

[29] Denzin NK, Lincoln YS (2011). The Sage handbook of qualitative research.

[30] Miles MB, Huberman AM, Saldana J (2013). Qualitative data analysis.

[31] Thorne S (2000). Data analysis in qualitative research. Evid-Based Nurs.

[32] Thorne S (2016). Interpretive description: qualitative research for applied practice.

[33] QSR International Pty Ltd. NVivo qualitative data analysis software. 10. ed2016.

[34] Russell E, Kosny A (2018). Communication and collaboration among return-to-work stakeholders. Disabil Rehabil.

[35] Jetha A, Lamontagne AD, Lilley R, Hogg-Johnson S, Sim M, Smith P (2017). Workplace social system and sustained return-to-work: a study of supervisor and co-worker supportiveness and injury reaction. J Occup Rehabil.

[36] Franche R-L, Krause N (2002). Readiness for return to work following injury or illness: conceptualizing the interpersonal impact of health care, workplace, and insurance factors. J Occup Rehabil.

[37] McLellan RK (2017). Work, health, and worker well-being: roles and opportunities for employers. Health Aff.

[38] Gensby U, Lund T, Kowalski K, Saidj M, Jørgensen AMK, Filges T (2012). Workplace disability management programs promoting return to work: a systematic review. Campbell Syst Rev.

[39] MacEachen E, Clarke J, Franche R-L, Irvin E (2006). Systematic review of the qualitative literature on return to work after injury. Scand J Work Environ Health.

[40] Dorfman L, Wallack L, Woodruff K (2005). More than a message: framing public health advocacy to change corporate practices. Health Educ Behav.

[41] Freimuth VS, Quinn SC (2004). The contributions of health communication to eliminating health disparities. Am J Public Health.

[42] Toll BA, O'Malley SS, Katulak NA, Wu R, Dubin JA, Latimer A (2007). Comparing gain-and loss-framed messages for smoking cessation with sustained-release bupropion: a randomized controlled trial. Psychol Addict Behav.

[43] Block LG, Keller PA (1995). When to accentuate the negative: the effects of perceived efficacy and message framing on intentions to perform a health-related behavior. J Mark Res.

[44] Noar SM, Benac CN, Harris MS (2007). Does tailoring matter? Meta-analytic review of tailored print health behavior change interventions. Psychol Bull.

[45] Wanyonyi KL, Themessl-Huber M, Humphris G, Freeman R (2011). A systematic review and meta-analysis of face-to-face communication of tailored health messages: implications for practice. Patient Educ Couns.

